# An Unusual Case of Spontaneous Esophageal Rupture after Swallowing a Boneless Chicken Nugget

**DOI:** 10.1155/2016/5971656

**Published:** 2016-02-02

**Authors:** Zeenia Aga, Jackie Avelino, Gail E. Darling, Jo Jo Leung

**Affiliations:** ^1^Faculty of Medicine, University of Toronto, Toronto, ON, Canada M5S 1A8; ^2^Department of Emergency Medicine, University Health Network, Toronto, ON, Canada M5G 2C4; ^3^Division of Thoracic Surgery, University Health Network, Toronto, ON, Canada M5G 2C4

## Abstract

A 25-year-old previously healthy man presented to our Emergency Department with shortness of breath and epigastric pain after swallowing a boneless chicken nugget one hour prior to presentation. Physical examination revealed epigastric rigidity and tenderness. Serology was normal except for mildly elevated bilirubin and amylase. Computed tomography (CT) scan of the chest revealed a distal esophageal rupture with accompanying pneumomediastinum and left-sided pleural effusion. Treatment was initiated with administration of intravenous fluids and broad-spectrum antibiotics. Subsequently, an esophageal stent was inserted endoscopically in addition to VATS (Video-Assisted Thoracoscopic Surgery) drainage of the left-sided pleural space. This case illustrates an unusual presentation of Boerhaave's syndrome: a rare and life-threatening form of noniatrogenic esophageal rupture most often preceded by forceful vomiting. Our case demonstrates that physicians should maintain an index of suspicion for spontaneous esophageal rupture in patients presenting with shortness of breath and epigastric pain even in the absence of preceding vomiting, cough, or seizure. Additionally, ingestion of boneless, shell-less foods may be sufficient to cause rupture in individuals without underlying esophageal pathology. CT scan of the thorax and upper abdomen should be performed in these patients to rule out this rare and life-threatening diagnosis.

## 1. Introduction 

Esophageal rupture is most commonly caused by accidental endoscopic perforation [1–4]. Rarely, spontaneous perforation of the esophagus can occur due to a rapid rise in intraluminal pressure after forceful vomiting in a phenomenon known as Boerhaave's syndrome. This occurs most often in the lateral, lower 1/3 of the esophagus [5, 6] and is associated with a mortality rate of 20–75% [7–9]. Many patients present with symptoms such as chest pain, shock, or respiratory distress and physical exam findings are often nonspecific (tachycardia, tachypnea, or fever). As a result of these nonspecific findings, Boerhaave's syndrome is often misdiagnosed as an aortic emergency, pericarditis, myocardial infarction, pulmonary embolus, spontaneous pneumothorax, perforated peptic ulcer, or pancreatitis [10–12]. Delayed diagnosis is one of the major differences in the management of iatrogenic esophageal rupture versus spontaneous rupture and may be responsible for the higher mortality rate in the latter. To our knowledge, this is the first published case of esophageal rupture following ingestion of a boneless, soft food item in the absence of any underlying esophageal pathology or food impaction [13], with only eight cases worldwide of spontaneous esophageal rupture without a preceding episode of vomiting, seizure, or prolonged cough [14]. We outline the case of a 25-year-old man, who presented to the ED with shortness of breath and epigastric pain starting immediately after swallowing a boneless chicken nugget. We will discuss the clinical presentation, appropriate diagnostic steps, and treatment strategies of this rare but potentially life-threatening condition.

## 2. Case Presentation

A 25-year-old previously healthy male presented to our Emergency Department with shortness of breath and epigastric pain that started immediately after swallowing a boneless chicken nugget one hour prior to presentation. He had no antecedent history of marked vomiting prior to symptom onset and denied any foreign body sensation. He had no significant past medical history with the exception of 2-3-week duration of mild gastroesophageal reflux. Our patient was not taking any medications at the time of presentation.

At admission, he was afebrile with a pulse of 64 beats/min, blood pressure of 134/87 mmHg, and oxygen saturation of 100%. He was alert and oriented with facial pallor and appeared uncomfortable on the stretcher. Physical examination revealed a tender, rigid abdomen (predominantly in the epigastric region) with voluntary guarding (2+) but no signs of peritonitis. On respiratory examination equal air entry was auscultated bilaterally with no adventitious sounds, no signs of subcutaneous air or tracheal deviation. He was able to speak clearly. Heart sounds were normal (S1, S2, and no murmurs) and no evidence of previous surgeries was found. The rest of the physical examination was unremarkable.

**Figure 1:**
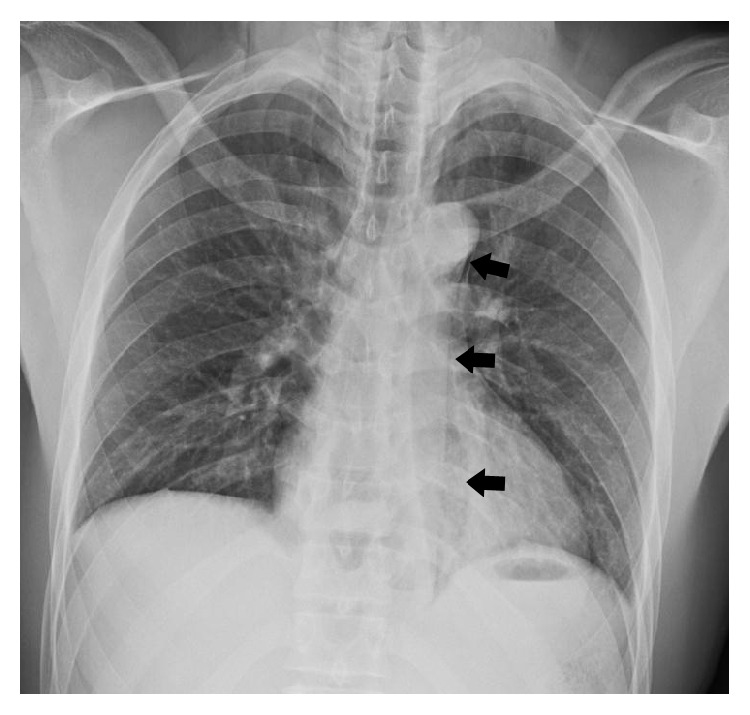
Chest X-ray displaying pneumomediastinum (indicated by black arrows) and left-sided pleural effusion.

**Figure 2:**
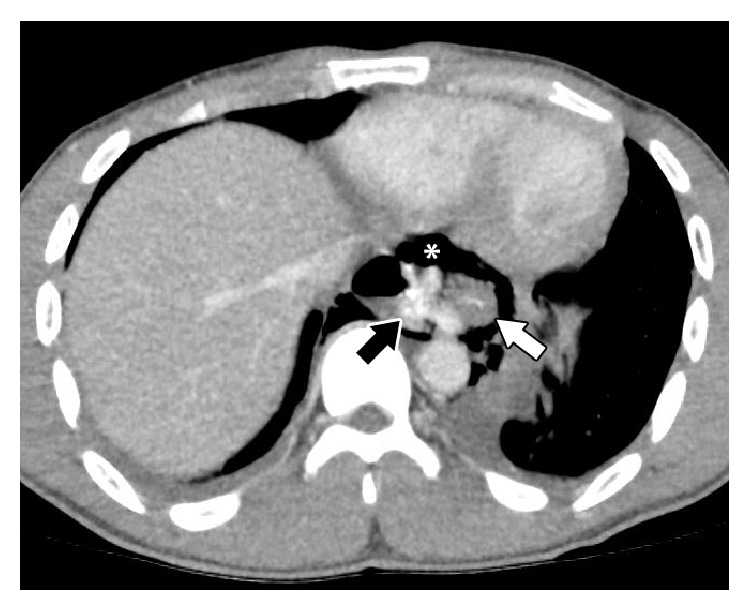
Chest computed tomography (CT) scan revealing a distal esophageal rupture with an oral contrast leak (indicated by the black arrow) from the ruptured esophagus (indicated by the white arrow), in addition to pneumomediastinum (asterisk) and left-sided pleural effusion.

Blood work was normal at the time of presentation (CBC, calcium, phosphate, magnesium, sodium, chloride, bicarbonate, anion gap, AST/ALT/ALP, and albumin) except for slight elevations of WBC (14.0 × 10^9^ cells/L), bilirubin (28 *μ*mol/L), and amylase (185 U/L). Lactate was tested six hours later, revealing an elevated level of 4.1 mmol/L. Chest X-ray was suggestive of mediastinal air (Figure 1) and revealed a left-sided pleural effusion. There was no evidence of foreign objects in the esophagus or free air in the abdomen. A computed tomography (CT) scan of the chest revealed a rupture in the distal esophagus with oral contrast leaking from the rupture, a pneumomediastinum, and left-sided pleural effusions (Figure 2).

Treatment began with cessation of oral intake, administration of IV fluids, morphine, and intravenous broad-spectrum antibiotics, and repeat blood work. As the patient was clinically well and did not require emergent surgery, he was admitted under the Thoracic Surgery Team and transferred to a step-down unit. Esophageal gastroscopy was performed revealing a mucosal tear at 40 cm into the esophagus, extending just to the gastroesophageal junction. An esophageal stent was positioned to cover the perforation. Video-Assisted Thoracoscopic Surgery (VATS) drainage of the pleural space was conducted with samples taken for culture. Intercostal nerve blocks were placed and a right-angle chest tube was inserted on the left side.

After 5 days, he developed a fever with increasing chest pain and leukocytosis whilst still on antibiotics. CT scan of the chest showed a mediastinal abscess and right pleural collection. He underwent VATS drainage of the abscess and decortication of the right pleural space. After three weeks, his esophageal stent was removed and the esophageal tear was observed to have healed nicely.

## 3. Discussion

The nonspecific physical exam findings and the lack of any classical symptoms of Boerhaave's syndrome often result in delayed and misdiagnosis of this rare and lethal form of noniatrogenic esophageal rupture. As such, it has a significant mortality rate estimated between 20 and 75% [7, 8]. “Mackler's Triad,” consisting of repeated vomiting (79%), lower chest pain (83%), and subcutaneous emphysema (27%), is only present in a minority of patients, and approximately half of all cases of Boerhaave's syndrome are atypical [10, 12, 15, 16]. Mediastinitis, sepsis, and shock are frequently seen late in the course of the illness, which further confuses the diagnostic picture. Given the low incidence of noniatrogenic esophageal rupture (less than 10%), it is unlikely for physicians to have a high index of suspicion for esophageal rupture compared to more common causes of chest pain, shortness of breath, and vomiting.

We report the case of a 25-year-old, previously healthy patient who developed a distal esophageal rupture and left-sided pleural effusion shortly after swallowing a boneless chicken nugget, during which he felt a sharp, scratching sensation. Importantly, our patient had no antecedent history of forceful vomiting (or similar esophageal strain through coughing, laughing, or seizure) which is typically associated with noniatrogenic rupture of the esophagus. At presentation, he showed nonspecific symptoms of dyspnea and epigastric pain and physical examination revealed a tender, rigid abdomen. Blood work was predominantly normal at presentation. It was only after a chest X-ray and CT scan of the chest that we were able to make a clear diagnosis of esophageal rupture. Importantly, there was no radiological evidence of an esophageal foreign body or food impaction, which can also cause spontaneous esophageal rupture [13].

This case demonstrates two salient teaching points for physicians and emergency responders. Firstly, noniatrogenic, life-threatening esophageal rupture can occur in the absence of any preceding history of vomiting, seizure, or chronic cough in patients without underlying esophageal pathology. Boerhaave's syndrome is classically described as a spontaneous transmural perforation of the esophagus associated with repetitive vomiting and is distinguished from a Mallory-Weiss tear based on the depth of esophageal damage. However, this case demonstrates that physicians should maintain an index of suspicion for Boerhaave's syndrome in patients presenting with shortness of breath and epigastric pain, even in the absence of a preceding history of strain through either vomiting, seizure, parturition, or chronic coughing and laughing. To our knowledge, this unique presentation has never been described in Canadian literature, with only eight published cases in the English literature [14, 17]. In these patients, CT of the chest and abdomen should be performed to rule out esophageal rupture. Second, and most unique to our case, is that even ingestion of boneless, shell-less, soft food items such as a chicken nugget may be sufficient to raise intraluminal pressure to the point of perforation in patients with no underlying esophageal pathology. To our knowledge, this is the first published case worldwide demonstrating esophageal rupture after swallowing a boneless, shell-less food item in a healthy esophagus without any radiological evidence of food impaction. Chicken nuggets have the consistency of many commonly eaten foods that are not typically associated with esophageal perforation (i.e., foods that are boneless and flexible and without sharp edges). Thus, physicians and emergency responders should not rule out esophageal perforation based on the consistency of the last meal and patients with suspicious findings for perforation including new epigastric pain or shortness of breath should be sent for radiological assessment.

The management of Boerhaave's syndrome, regardless of the specific cause, begins with cessation of oral intake, administration of intravenous fluids and broad-spectrum antibiotics followed by surgical or endoscopic treatment of the tear [18–21]. An isolated nonoperative approach can only be taken in a minority of patients who have radiologic findings showing lack of mediastinal or pleural contamination and no systemic symptoms of infection at the time of presentation [9, 11, 22, 23].

## 4. Conclusion

Our case illustrates the need for emergency physicians to consider the rare but life-threatening diagnosis of spontaneous esophageal rupture in patients presenting with epigastric pain and shortness of breath without a preceding history of forceful vomiting or persistent cough. Additionally, physicians should consider the possibility of esophageal perforation induced by forcefully swallowing food items, even in the case of boneless, shell-less, soft foods in patients with no underlying esophageal disease.

## References

[1] Vidarsdottir H., Blondal S., Alfredsson H., Geirsson A., Gudbjartsson T. (2010). Oesophageal perforations in Iceland: a whole population study on incidence, aetiology and surgical outcome. *The Thoracic and Cardiovascular Surgeon*.

[2] Merchea A., Cullinane D. C., Sawyer M. D. (2010). Esophagogastroduodenoscopy-associated gastrointestinal perforations: a single-center experience. *Surgery*.

[3] Gupta N. M., Kaman L. (2004). Personal management of 57 consecutive patients with esophageal perforation. *The American Journal of Surgery*.

[4] Bladergroen M. R., Lowe J. E., Postlethwait R. W. (1986). Diagnosis and recommended management of esophageal perforation and rupture. *The Annals of Thoracic Surgery*.

[5] De Schipper J. P., Pull Ter Gunne A. F., Oostvogel H. J. M., Van Laarhoven C. J. H. M. (2009). Spontaneous rupture of the oesophagus: Boerhaave's syndrome in 2008. Literature review and treatment algorithm. *Digestive Surgery*.

[6] Hill A. G., Tiu A. T., Martin I. G. (2003). Boerhaave's syndrome: 10 years experience and review of the literature. *ANZ Journal of Surgery*.

[7] Janjua K. J. (1997). Boerhaave's syndrome. *Postgraduate Medical Journal*.

[8] Connelly C. L., Lamb P. J., Paterson-Brown S. (2013). Outcomes following Boerhaave's syndrome. *Annals of the Royal College of Surgeons of England*.

[9] Wahed S., Dent B., Jones R., Griffin S. M. (2014). Spectrum of oesophageal perforations and their influence on management. *British Journal of Surgery*.

[10] van der Weg G., Wikkeling M., van Leeuwen M., ter Avest E. (2014). A rare case of oesophageal rupture: Boerhaave's syndrome. *International Journal of Emergency Medicine*.

[11] Granel-Villach L., Fortea-Sanchis C., Martínez-Ramos D. (2014). Boerhaave's syndrome: a review of our experience over the last 16 years. *Revista de Gastroenterología de México*.

[12] Henderson J. A., Péloquin A. M. (1989). Boerhaave revisited: spontaneous esophageal perforation as a diagnostic masquerader. *The American Journal of Medicine*.

[13] Aronberg R. M., Punekar S. R., Adam S. I., Judson B. L., Mehra S., Yarbrough W. G. (2015). Esophageal perforation caused by edible foreign bodies: a systematic review of the literature. *The Laryngoscope*.

[14] Kamiyoshihara M., Kakinuma S., Kusaba T. (1998). Occult Boerhaave's syndrome without vomiting prior to presentation. Report of a case. *The Journal of Cardiovascular Surgery*.

[15] Blencowe N. S., Strong S., Hollowood A. D. (2013). Spontaneous oesophageal rupture. *British Medical Journal*.

[16] Lemke T., Jagminas L. (1999). Spontaneous esophageal rupture: a frequently missed diagnosis. *The American Surgeon*.

[17] Søreide J. A., Viste A. (2011). Esophageal perforation: diagnostic work-up and clinical decision-making in the first 24 hours. *Scandinavian Journal of Trauma, Resuscitation and Emergency Medicine*.

[18] Sepesi B., Raymond D. P., Peters J. H. (2010). Esophageal perforation: surgical, endoscopic and medical management strategies. *Current Opinion in Gastroenterology*.

[19] Ben-David K., Behrns K., Hochwald S. (2014). Esophageal perforation management using a multidisciplinary minimally invasive treatment algorithm. *Journal of the American College of Surgeons*.

[20] Mavroudis C. D., Kucharczuk J. C. (2014). Acute management of esophageal perforation. *Current Surgery Reports*.

[21] Schweigert M., Beattie R., Solymosi N. (2013). Endoscopic stent insertion versus primary operative management for spontaneous rupture of the esophagus (Boerhaave syndrome): an international study comparing the outcome. *The American Surgeon*.

[22] Zhao G.-J., Cheng J.-Y., Zhi S.-C., Jin X., Lu Z.-Q. (2015). Conservative management of esophageal perforation due to external air-blast injury: a case report and literature review. *Therapeutic Advances in Gastroenterology*.

[23] Peng A., Li Y., Xiao Z., Wu W. (2012). Study of clinical treatment of esophageal foreign body-induced esophageal perforation with lethal complications. *European Archives of Oto-Rhino-Laryngology*.

